# Unintentional injection of contrast agent into the subdural space during an epidural blood patch procedure identified the site of cerebrospinal fluid leak: a case report

**DOI:** 10.1186/s40981-025-00830-8

**Published:** 2025-11-28

**Authors:** Momoko Hattori, Hiroai Okutani, Mayuu Kobata, Daisuke Ishimoto, Yumiko Takao, Nobutaka Kariya, Munetaka Hirose

**Affiliations:** 1 Department of Anesthesiology, Hyogo Medical University, 1-1, Mukogawa-Cho, Nishinomiya, Hyogo 663-8501 Japan; 2 Department of Pain Clinic, Hyogo Medical University Hospital, 1-1, Mukogawa-Cho, Nishinomiya, Hyogo 663-8501 Japan

**Keywords:** Cerebrospinal fluid leak, Epidural blood patch, Subdural space

## Abstract

**Background:**

CT myelography is a definitive diagnostic tool for cerebrospinal fluid leakage. We report a case in which the site of a cerebrospinal fluid leak that had been difficult to identify on CT myelography was fortuitously localized owing to incidental injection of a contrast agent into the subdural space.

**Case presentation:**

A 37-year-old man developed headache and neck pain following a massage. He was diagnosed with intracranial hypotension, but the exact site of cerebrospinal fluid leakage could not be identified by CT myelography. An epidural blood patch was performed at the Th11/12 level, during which a contrast agent was inadvertently injected into the subdural space, revealing cerebrospinal fluid leakage from the Th2/3 level into the epidural space.

**Conclusions:**

An unintentional subdural injection of contrast agent resulted in extensive distribution, facilitating the precise identification of the cerebrospinal fluid leakage site.

## Background

Epidural blood patch (EBP) therapy for cerebrospinal fluid (CSF) leakage has been reported to improve outcomes in approximately 40–70% of patients [1, 2]. The therapy involves the precise injection of autologous blood into the epidural space. We present a case in which the site of spinal CSF leakage, which could not be detected by CT myelography, was fortuitously identified during an EBP procedure when contrast medium was inadvertently injected into the subdural space.

## Case presentation

A 37-year-old man (height 159 cm, weight 63 kg, BMI 24.7 kg/m^2^) with no significant past medical or family history presented with sudden-onset headache and posterior neck pain a few days after receiving a massage. He was transported to a local emergency department, where brain MRI revealed no remarkable findings (including dural enhancement or thickening). His symptoms temporarily improved with bed rest and intravenous fluid administration, but recurrent and persistent headaches prompted a referral to our institution 30 days after onset for further evaluation and treatment.

**Fig. 1:**
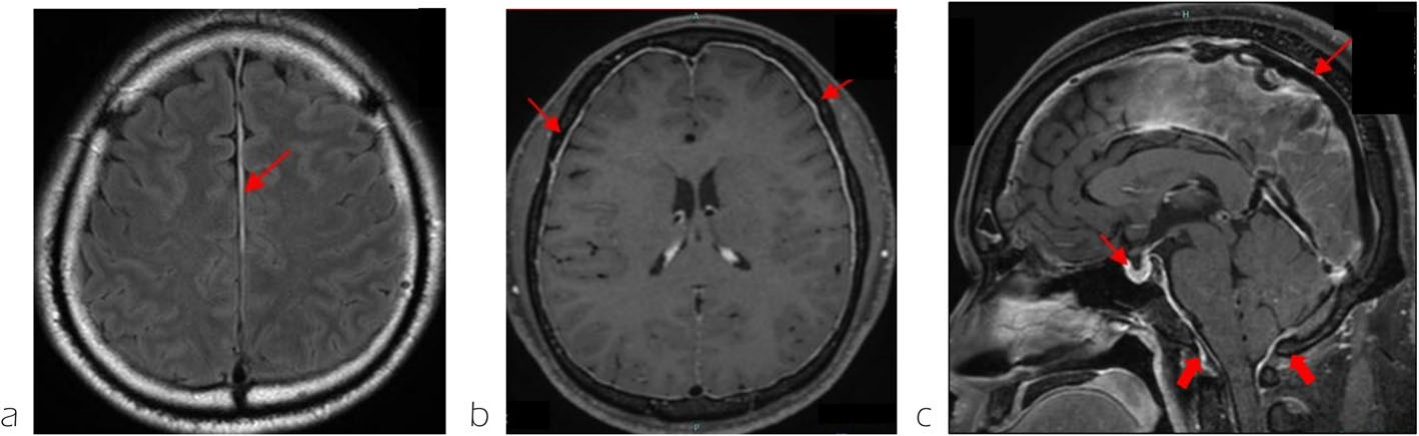
Subdural hematoma characteristics. **a** a subdural hematoma was identified around the falx cerebri (arrow). **b** Dural thickening was observed (arrows). **c** Enlargement of the pituitary gland, brain sagging and dilation of the venous sinus were noticed (arrows)

**Fig. 2:**
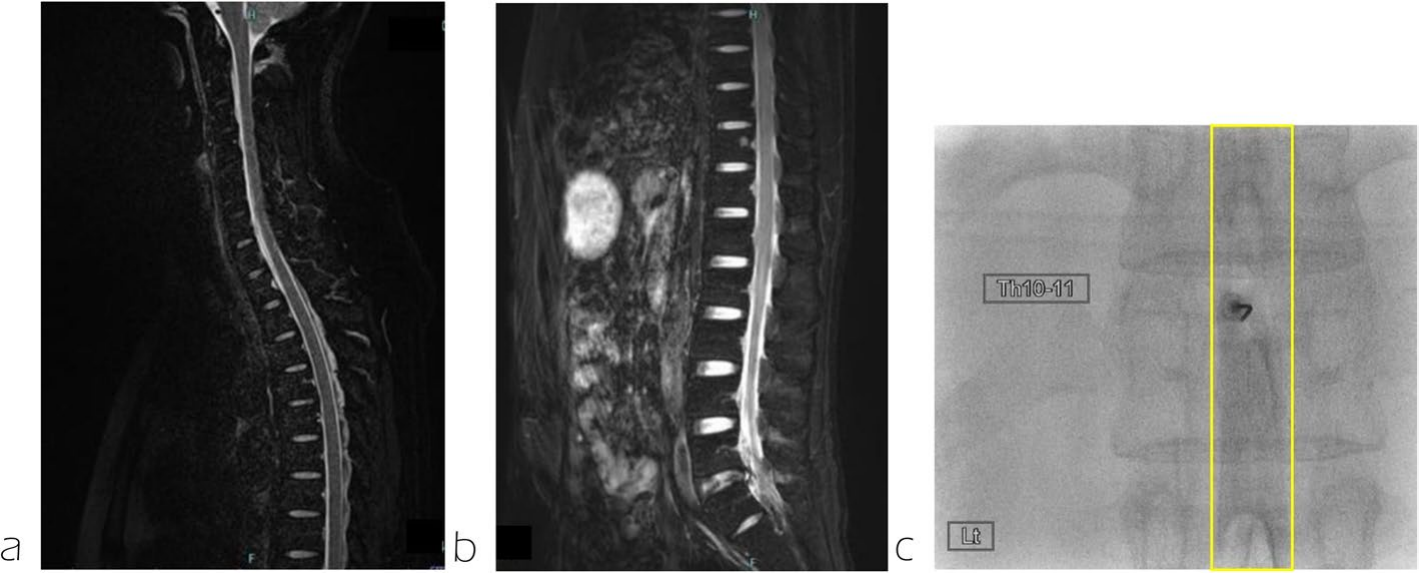
Spinal MRI, CT myelography and fluoroscopy results. **a** High-intensity signals were noted within the epidural space on fat-suppressed T2-weighted spinal MRI. **b** Epidural contrast enhancement observed on CT myelography. **c** Subdural contrast distribution was identified on fluoroscopy (square)

**Fig. 3:**
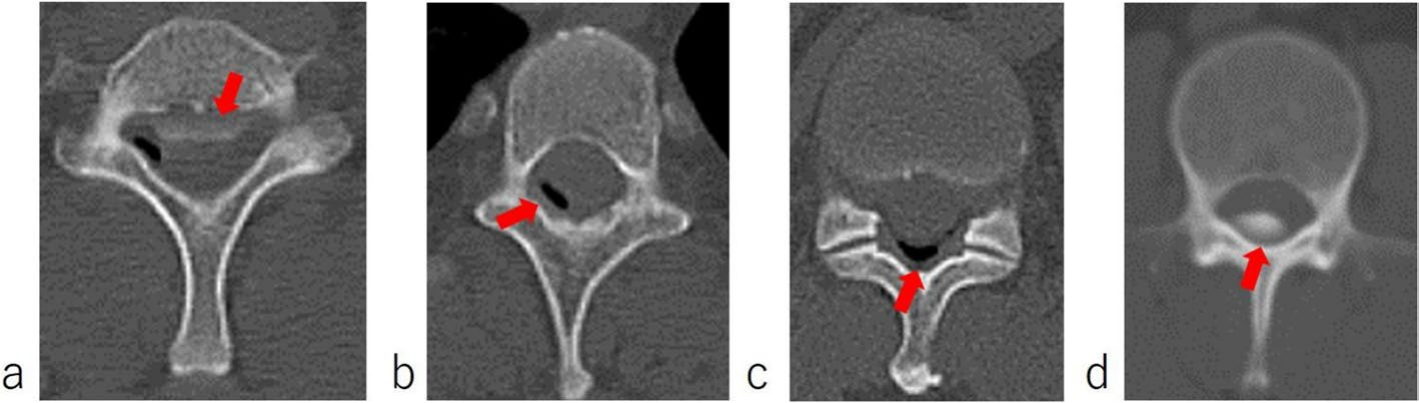
Post procedural CT results (**a**) Contrast agent accumulation in the ventral epidural space at the level of the second thoracic vertebra(arrow). **b** Air retention in the epidural space at the level of the third thoracic vertebra (arrow). **c** Air retention in the epidural space at the level of the 12th thoracic vertebra (arrow). **d** Contrast agent accumulation in the subdural space below the level of the 12th thoracic vertebra (arrow)

**Fig. 4:**
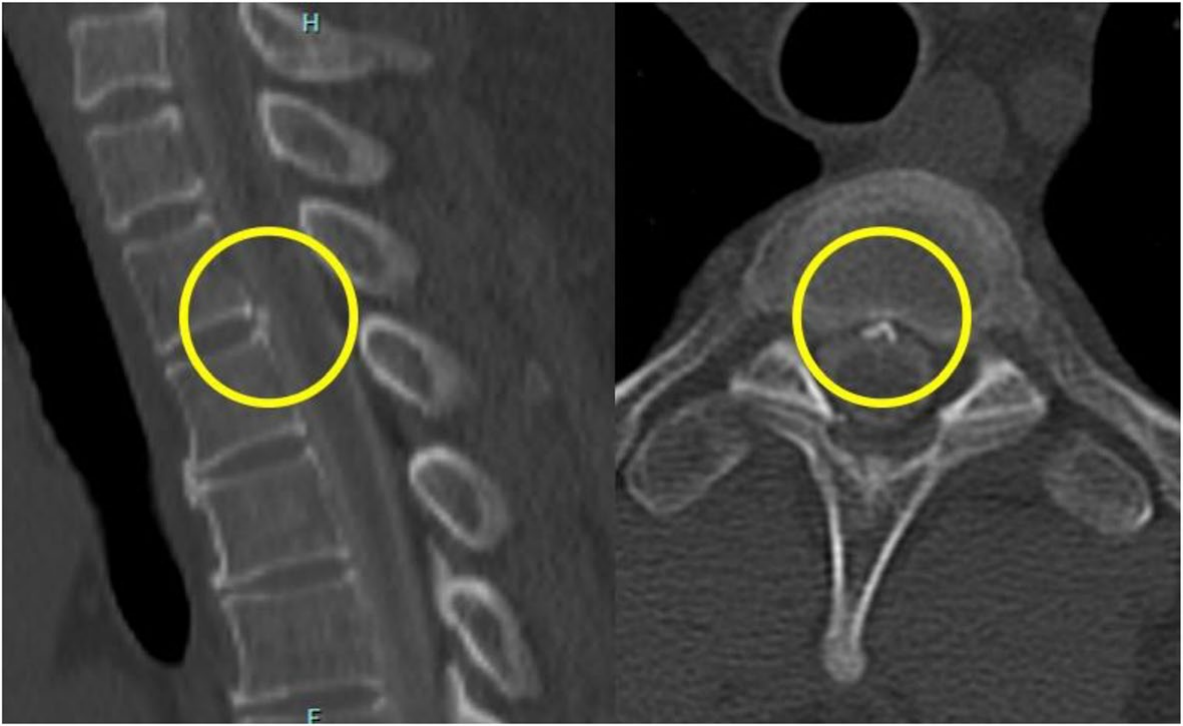
Osteophyte formation at Th2/3. Bone spurs were present on the second and third thoracic vertebrae (circles)

No neurological deficits were observed at admission. The patient reported right temporal-to-posterior neck pain, which became more acute in the upright position and resolved in the supine position. Brain MRI revealed diffuse dural thickening, pituitary gland enlargement, brain sagging, and venous sinus distention. A subdural hematoma was also observed in the cerebral falx region (Fig. 1a-c). No abnormal signals were detected in the brain parenchyma. These findings were consistent with a diagnosis of intracranial hypotension due to CSF leakage. Spinal MRI and CT myelography were performed to identify the CSF leakage site. Although CSF leakage was observed around the thoracic spine, the exact site of the leakage could not be determined (Fig. 2a, b). The patient was placed in a prone position, and a 22G nerve block needle was inserted at the Th11/12 interspace. The procedure was performed under fluoroscopic guidance using the loss-of-resistance technique with saline and air injections. The resistance of the glass syringe originally employed for the technique was particularly high. To facilitate the loss-of-resistance determination, a small amount of air was injected in addition to saline. However, since the resistance remained difficult to determine, the syringe was replaced to resolve the issue. After confirming loss of resistance and absence of CSF backflow, contrast medium (Iohexol) was injected, resulting in subdural spread (Fig. 2c). The needle was slightly withdrawn until the loss of resistance was reconfirmed, and the position of the needle tip was rechecked using contrast medium, which showed slight visualization of the nerve root region. Administration was discontinued when the patient complained of back pain after 13 ml of autologous blood and 5 ml of contrast agent had been injected. A CT scan performed immediately afterwards revealed that almost all of the mixture had penetrated into the subdural space. The patient was placed on bed rest for 3 h after receiving the injection, and reported experiencing pain that extended from the occipital region to the neck. A CT scan performed 3 h later revealed contrast agent spread in the ventral epidural space from the cervical spine to the Th2 level (Fig. 3a), and air accumulation in the dorsal epidural space at the Th3 level (Fig. 3b). No contrast agent retention was observed between the Th3 and Th11 levels; however, air was observed in the epidural space at the Th12 level (Fig. 3c). Below the Th12 level, presence of contrast agent was observed in the dorsal subdural space (Fig. 3d). A review of the spinal CT images revealed osteophyte formation at Th2/3 (Fig. 4), which corresponded to the suspected site of CSF leakage. We concluded that autologous blood injected into the subdural space at the Th11/12 level ascended and leaked into the epidural space through a CSF fistula at the Th3 level.

**Fig. 5:**
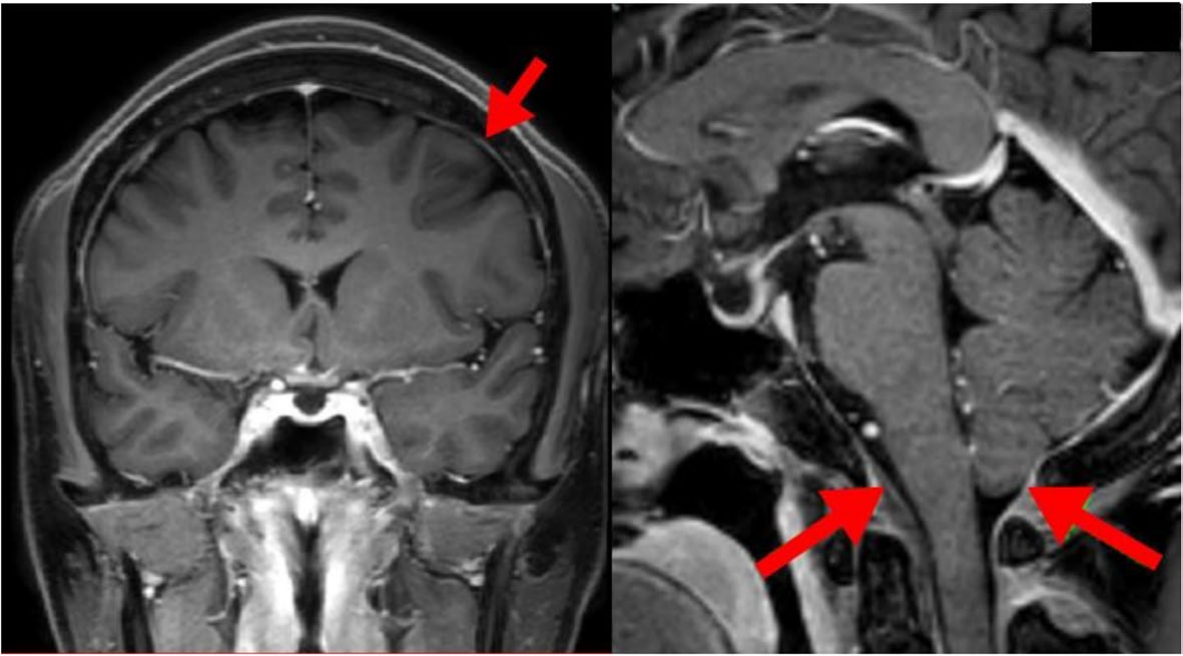
Follow-up brain MRI results. Brain sagging and dural thickening improved after treatment (arrows)

After the procedure, the symptoms of headache had markedly resolved after 24 h. The patient recovered uneventfully and was discharged on postoperative day 6. Follow-up brain MRI performed after one month showed resolution of the brain sagging and diffuse dural thickening (Fig. 5).

## Discussion

This case highlights three important clinical features. First, the site of CSF leakage, which could not be identified by CT myelography, was incidentally localized through an unintended subdural injection of autologous blood with contrast agent during EBP therapy. Second, although autologous blood was delivered into the subdural space rather than into the epidural space, the primary therapeutic goal of sealing the CSF leak was achieved. Third, fluoroscopic guidance during EBP is a valuable tool to ensure accurate delivery into the epidural space and to identify procedural irregularities in real-time.

In this particular case, autologous blood with contrast agent was inadvertently injected into the subdural space during the EBP procedure, leading to identification of the CSF leak site. The subdural space is not a true anatomical space under normal physiological conditions; rather, it is considered a potential space that may form transiently between the dura mater and arachnoid membrane following mechanical stimuli such as needle insertion or fluid injection. Owing to its extremely narrow anatomy, even a small volume of the injected fluid can spread widely within this space [3]. In our case, despite the significant distance between the puncture site (Th11/12) and the leakage site (Th2/3), both localization of the CSF leak and symptom relief were achieved. This outcome suggests that the injected agent traveled upward and diffused over a broad area in the subdural space, eventually covering and sealing the leakage site. The absence of contrast in the subdural space between Th3 and Th11 could be attributed to the timing of the CT scan, which was performed 3 h after the EBP procedure. The increase in subdural pressure following the injection may have caused the contrast agent to leak through a dural defect during the interval before imaging. It was later revealed that the patient had received a vigorous neck massage prior to headache onset. CT imaging revealed osteophyte formation at the Th2/3 level, which may have contributed to the dural injury and subsequent CSF leakage triggered by external mechanical stress. Although CT myelography is a valuable diagnostic tool for identifying CSF leak sites, it failed to localize the leak precisely in this particular case, although CSF extravasation was confirmed. In many previous studies using conventional imaging, the thoracolumbar spine was the predominant site of CSF leakage [4]. Considering the potential involvement of the upper thoracic spine due to the neck massage, puncture at a higher thoracic level could have represented an appropriate option. At the time of EBP, no information was available suggesting the previous application of significant force to the cervical region. Considering that spontaneous intracranial hypotension is most commonly reported at the thoracic level in systematic reviews, and that the lower thoracic region allows for easier access to perform a puncture, EBP was performed at a lower vertebral level [2, 5].

Fluoroscopic guidance is useful for ensuring accurate injection into the epidural space of the spine. In a large retrospective clinical series that included 628 patients, fluoroscopic guidance was used to categorize contrast patterns for epidural, subdural, and combined injections, and the respective incidences were as follows: 49% for epidural, 19.6% for subdural, and 31% for combined injections. The subdural pattern was associated with a higher incidence and longer duration of motor block, supporting the routine use of contrast fluoroscopy to identify the space and avoid complications [4].

In our case, post-procedural imaging revealed that the injection entered the subdural space, indicating that the needle tip was likely positioned across both the subdural and epidural spaces. When a contrast agent is injected into the subdural space, it typically appears as a thin elongated contour localized to the dorsal aspect of the spinal canal, often demonstrating pulsatile movement synchronized with the cardiac cycle. Contrast agents injected into the epidural space delineate the outlines of nerve roots and ganglia [3]. Therefore, fluoroscopic guidance increases the likelihood of accurate epidural placement by allowing real-time visualization of these distinctive imaging features.

In the present case, the contrast agent was inadvertently injected into the subdural space, which subsequently enabled the identification of the CSF leak site. However, unintentional subdural injection during EBP performed without fluoroscopic guidance is common, and several cases have been reported. For example, in a case of subdural hematoma after an EBP, the patient developed severe low back pain following blind EBP. Subsequent imaging revealed a subdural hematoma, indicating that autologous blood was injected into the subdural space of the patient [6]. This case is valuable because it demonstrates that EBP can be effective even when the injection site is distant from the CSF leak site.

In conclusion, we encountered a case of CSF leakage in which the leakage site, which could not be detected using standard diagnostic tools such as CT myelography, was successfully identified owing to the unintentional injection of a contrast agent into the subdural space, and furthermore, improvement in symptoms was achieved. This represents a rare case demonstrating that, despite the injection pathway penetrating into the subdural space, the therapeutic goal was ultimately achieved. This experience suggests that fluoroscopy-guided blood patching with contrast may contribute to a better understanding CSF dynamics, the visualization of leakage sites, and even symptom improvement. Therefore, fluoroscopy-guided blood patch should be considered a useful technique possessing both diagnostic value and therapeutic significance.

## Data Availability

Not applicable.

## Abbreviations

CSF: Cerebrospinal fluid
EBP: Epidural blood patch
